# Psychological factors associated with Chinese users’ adoption of AI-generated health education short videos: a PLS-SEM–ANN–IPMA study

**DOI:** 10.3389/fpsyg.2026.1889095

**Published:** 2026-09-23

**Authors:** Jun Liu, Xinyun Liang, Yue Sun, Ying Hu, Linxin Liu

**Affiliations:** 1 College of Art, Cheongju University, Cheongju, Republic of Korea; 2 School of Literature and Communication, Sichuan University of Arts and Science, Dazhou, China; 3 School of Design, Fujian University of Technology, Fuzhou, China; 4 Department of Critical Care Medicine (Surgery), Mengchao Hepatobiliary Hospital of Fujian Medical University, Fuzhou, China

**Keywords:** AI trust, AI-generated health education short videos, artificial neural network, health anxiety, information adoption model, IPMA, PLS-SEM

## Abstract

**Background:**

AI-generated health education short videos are becoming an important form of digital health communication. Compared with conventional health education content, these videos require users to evaluate not only the value of health information, but also the credibility of information sources, the trustworthiness of AI-generated content, and their own health-related psychological state. Therefore, it is necessary to further examine the psychological factors that influence users’ adoption of AI-generated health information.

**Methods:**

Drawing on the Information Adoption Model, this study developed a research framework that includes information quality, source credibility, perceived usefulness, AI trust, health anxiety, and information adoption intention. A questionnaire survey was conducted among Chinese users, yielding 476 valid responses. The data were analyzed using partial least squares structural equation modeling, artificial neural networks, and importance–performance map analysis.

**Results:**

The PLS-SEM results showed that information quality and source credibility were significantly and positively associated with both perceived usefulness and AI trust. Perceived usefulness, AI trust, and health anxiety were also significantly and positively associated with information adoption intention. Among these factors, AI trust showed the strongest association with information adoption intention, whereas health anxiety was statistically significant but had a relatively weak effect. The mediation analysis further indicated that perceived usefulness and AI trust played important mediating roles between information-related factors and information adoption intention. The ANN results also identified AI trust as the most important predictor of information adoption intention. The IPMA results further showed that AI trust had the highest importance but a relatively insufficient performance level.

**Conclusion:**

This study extends the Information Adoption Model to the context of AI-generated health education short videos and highlights the central role of AI trust in users’ information adoption process. The findings suggest that improving transparency, strengthening source credibility, providing evidence-based content, and introducing expert review mechanisms may help enhance users’ trust in AI-generated health information and further increase their information adoption intention.

## 1 Introduction

Health is a fundamental basis for individual well-being, quality of life, and social development. Improving public health literacy and health information communication has become an important issue in health governance and health promotion (Caeiros et al., 2024; Liu et al., 2020). Health communication plays an important role in disease prevention, health education, and informed decision-making (Ishikawa and Kiuchi, 2010). With the rapid development of internet platforms and intelligent media technologies, the ways in which the public accesses health information are changing, increasingly characterized by video-based, convenient, and high-frequency information exposure (Schillinger et al., 2020; Tsao et al., 2021). The popularity of short-video platforms has reduced the temporal and spatial constraints of health knowledge dissemination and has further enhanced the accessibility, expressiveness, and everyday reach of health information (Yu et al., 2025). Against this background, health education short videos have become an important object of study in digital health communication research (Zhu et al., 2023). As a comprehensive form of health information communication, health education short videos involve not only health information content and professionalism but also information presentation, interaction, and user experience (Li et al., 2021; Xuan et al., 2024). With the entry of generative artificial intelligence into health communication, health education content that is fully or partially generated by AI is becoming a new resource through which users access and understand health information (Svestkova et al., 2025; Tilton et al., 2025). Generative AI can produce user-oriented health education content, explain complex medical information, and provide lifestyle suggestions, thereby supporting the production of content related to health knowledge interpretation, health management advice, and lifestyle guidance (Traylor et al., 2025). Unlike conventional health education short videos, AI-generated health education short videos require users to evaluate not only the value of the health information itself but also the generation process, information sources, and credibility of the content.

Existing studies have examined users’ acceptance and adoption of health information from multiple perspectives. First, research on health-related short videos has shown that short videos have become an important channel through which the public accesses health information and may influence users’ health knowledge, attitudes, intentions, and behaviors (Zhu et al., 2023). Second, studies on the adoption of health short videos have found that information quality, perceived usefulness, and perceived trust significantly affect users’ intention to adopt health short videos. This suggests that users’ adoption of health short videos is not determined merely by exposure or viewing behavior, but is closely related to their judgments about content value and credibility (Han et al., 2025). In addition, research on generative AI health information has begun to examine users’ trust in and willingness to use AI-generated health information, indicating that AI is becoming a new source of digital health information (Svestkova et al., 2025). These studies provide an important basis for understanding health information adoption and suggest that information value judgments, source credibility, and AI trust may be important factors influencing users’ adoption.

However, existing research still does not fully explain the mechanism underlying users’ information adoption of AI-generated health education short videos. On the one hand, previous studies have mainly focused on general health short videos or text-based generative AI health information tools, while paying limited attention to AI-generated health education short videos as an object that combines both video communication and AI generation (Han et al., 2025; Xie et al., 2024; Yu et al., 2025). Compared with general health-related short videos, AI-generated health education short videos involve not only users’ judgments about whether the health information is clear, useful, and easy to understand, but also their overall evaluation of the AI generation process, information sources, and content reliability. Therefore, this type of content should not be simply equated with ordinary health-related short videos or general AI health information tools; rather, it needs to be examined within a specific information adoption context.

Second, in terms of theoretical explanation, existing studies have more often examined users’ exposure to, use of, and responses to related content or tools. Less attention has been paid to whether health information presented in videos is actually accepted, referenced, and adopted by users from a perspective that integrates information adoption with individual psychological factors, especially the role of psychological factors such as health anxiety (Han et al., 2025; Wu et al., 2025). The Information Adoption Model (IAM) emphasizes the roles of information quality, source credibility, and perceived usefulness in information adoption, thereby providing an important theoretical foundation for this study. However, in the context of AI-generated health education short videos, users not only evaluate whether the information is useful, but also judge whether AI-generated content is reliable, trustworthy, and dependable. At the same time, health issues are highly personal and risk-related, and users may be influenced by health concerns, perceived disease risk, and self-protection needs when processing such information. Therefore, it is necessary to further incorporate AI trust and health anxiety into the IAM to better explain the adoption process of AI-generated health information.

Methodologically, PLS-SEM can be used to test the theoretical paths among variables in the extended IAM and assess their significance. To further examine whether the importance ranking of key predictors remains stable within a complementary predictive framework, this study introduces artificial neural networks (ANN) as an auxiliary analytical tool (Xu et al., 2023). It should be noted that ANN is not used in this study to replace PLS-SEM, nor is it intended to demonstrate complex nonlinear mechanisms. Instead, it is used to provide data-driven supplementary validation of the relative importance of significant predictors. In addition, importance–performance map analysis (IPMA) is used to identify practical factors that have high importance but still show room for improvement (Garg et al., 2025). Thus, PLS-SEM, ANN, and IPMA, respectively, serve the purposes of theoretical path testing, predictive robustness checking, and practical priority identification.

In response to the above research gaps, this study defines users’ evaluation of AI-generated health education short videos as an information adoption issue rather than merely a matter of media exposure or technology use. Based on the IAM, this study develops a research framework that includes information quality, source credibility, perceived usefulness, AI trust, health anxiety, and information adoption intention. Overall, this study makes three main contributions. First, it focuses on AI-generated health education short videos as an emerging form of health communication that combines video-based communication and AI-generated content, thereby expanding the research scope of digital health communication and health information adoption. Second, by incorporating AI trust and health anxiety into the IAM, this study contextualizes the traditional “information cues–perceived usefulness–information adoption” pathway. Specifically, information quality reflects users’ judgments about the content itself, source credibility reflects users’ judgments about the publisher or information source, and AI trust reflects users’ judgments about the reliability, trustworthiness, and dependability of AI-generated health information. In this way, the study further distinguishes content cues, source cues, and trust judgments related to AI generation, thereby enhancing the applicability of the IAM in the context of AI-based health communication. Third, this study combines PLS-SEM, ANN, and IPMA for theoretical path testing, predictive robustness checking, and practical priority identification, respectively. The findings provide guidance for improving the content quality of AI-generated health education short videos, strengthening source credibility, cultivating user trust, and developing platform governance strategies.

## 2 Literature review and hypothesis development

### 2.1 The information adoption model and its extension

The Information Adoption Model (IAM) is an important theoretical framework for explaining how individuals accept and adopt external information (Song et al., 2021). Proposed by Sussman and Siegal, the model integrates ideas from the Elaboration Likelihood Model (ELM) and the Theory of Reasoned Action (TRA). It emphasizes that, after being exposed to external information, individuals form value judgments by evaluating different information cues and then develop adoption intentions (Sussman and Siegal, 2003). In the classic IAM, information quality and source credibility are regarded as two core antecedents, while perceived usefulness serves as the key mechanism linking these antecedents to information adoption (Cheung et al., 2008; Elwalda et al., 2022). In other words, individuals’ evaluations of information content and sources influence their judgments about whether the information is helpful and valuable, which in turn affects information adoption (Erkan and Evans, 2016; Sussman and Siegal, 2003). Therefore, the core logic of the IAM can be summarized as follows: information cues influence perceived usefulness, and perceived usefulness further influences information adoption (Sussman and Siegal, 2003).

The theoretical strength of the IAM lies in its direct focus on how information is adopted, rather than on general technology acceptance or media use behavior (Mensah et al., 2022; Sun et al., 2019). Based on this feature, the IAM has been widely applied in research contexts such as online reviews (Salehi-Esfahani et al., 2016), electronic word-of-mouth (Erkan and Evans, 2016; Song et al., 2021), social media information (Elwalda et al., 2022; Shu and Scott, 2014), and digital health information (Jin et al., 2016). These studies use the model to explain how users form judgments about information value based on content cues and source cues. However, the classic IAM was mainly developed for general information communication contexts, and its core variables primarily focus on information characteristics and source evaluation (Erkan and Evans, 2016). When the information environment involves more complex sources of uncertainty, relying only on the original IAM variables may not fully explain users’ adoption judgments (Le, 2023). Therefore, previous studies have begun to introduce context-specific variables while retaining the core logic of the IAM, in order to improve the explanatory power of the model (Elwalda et al., 2022; Erkan and Evans, 2016; Khan et al., 2025; Salehi-Esfahani et al., 2016).

For the present study, although the original IAM can explain the basic relationships among information quality, source credibility, and perceived usefulness (Mensah et al., 2022), it is still insufficient to fully capture users’ adoption mechanism in the context of AI-generated information. This is because, in AI-generated content contexts, users not only evaluate whether the information is useful but also further judge whether it is credible and dependable (Afroogh et al., 2024; Gillath et al., 2021). Specifically, information quality mainly reflects users’ judgments about the clarity, completeness, and reference value of the content itself; source credibility mainly reflects users’ judgments about the professionalism and authority of the publisher or information source; and AI trust further reflects users’ judgments about the reliability, trustworthiness, and dependability of AI-generated health information. Therefore, it is necessary to incorporate additional variables into the IAM that can reflect trust judgments toward AI-generated content and individual psychological differences, so as to more comprehensively explain users’ information adoption process.

Based on this logic, this study extends the classic IAM by introducing two additional dimensions: AI trust and health anxiety. First, AI trust refers to users’ overall judgment of whether AI-generated information is reliable, credible, and dependable. It is an important psychological mechanism linking information evaluation and information adoption (Liu and Sun, 2025; Stevens and Stetson, 2023). Second, health anxiety reflects users’ individual psychological state and risk sensitivity, which may further influence their attention to information, risk judgment, and adoption tendency (Svestkova et al., 2025). Recent studies on virtual reality learning and generative AI use also indicate that, in emerging technology contexts, classic acceptance or adoption models often need to be extended by incorporating specific technological features and users’ psychological states in order to improve their explanatory power in specific contexts (Chiang et al., 2026; Yao et al., 2026). Accordingly, the “information cues–perceived usefulness–information adoption” pathway emphasized in the original IAM is further extended into an explanatory framework that simultaneously incorporates content cues, source cues, trust judgments toward AI-generated content, and individual differences in health-related psychological states.

Based on the above discussion, this study adopts the extended IAM as the overall theoretical framework, as shown in Figure 1. This framework retains the core logic of the IAM regarding information quality, source credibility, perceived usefulness, and information adoption. At the same time, it incorporates AI trust to explain how users’ trust judgments toward AI-generated health information are associated with information evaluation and information adoption intention. It also includes health anxiety to supplement the explanation of the information adoption process from the perspective of users’ health-related psychological states. In this way, this study develops an information adoption model that is more suitable for the context of AI-generated health education short videos and, on this basis, proposes hypotheses regarding the relationships among the variables.

**Figure 1:**
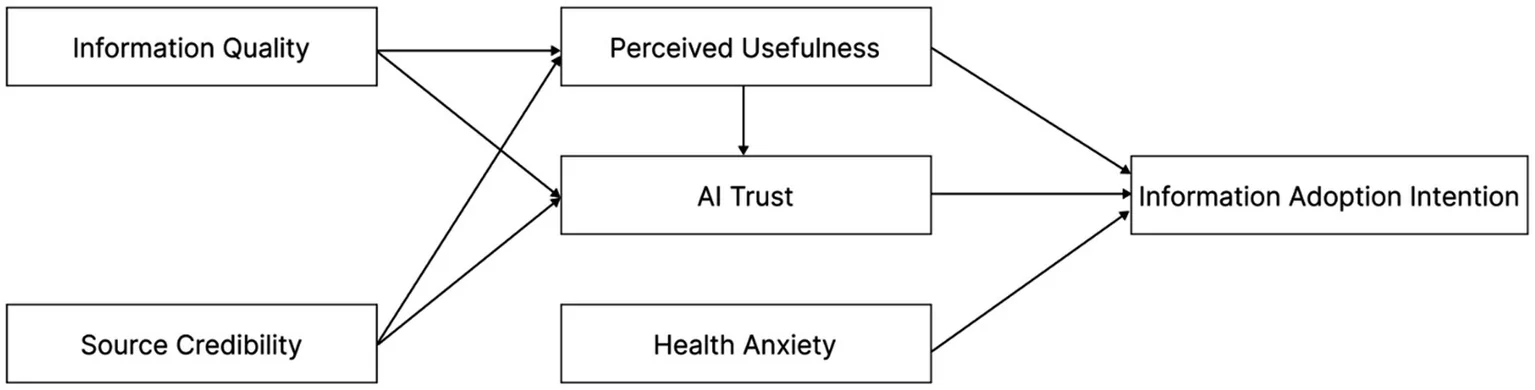
Conceptual model.

### 2.2 Research hypotheses

#### 2.2.1 Information quality

Information quality refers to the persuasiveness and attributes of the information itself, and it is an important criterion for users when judging and adopting information (Cheung et al., 2012). In general, information quality includes aspects such as accuracy, completeness, relevance, understandability, and effectiveness, and it is one of the most basic and important criteria for evaluating information resources (Stvilia, 2006; Xie et al., 2024). From the user perspective, information quality reflects the extent to which information meets users’ needs and provides reference value. When users perceive information quality to be high, they are more likely to form positive cognitive evaluations and value judgments about the information (Wu et al., 2025). In this study, information quality specifically refers to users’ subjective evaluation of the accuracy, clarity, completeness, relevance, and professionalism of the health information presented in AI-generated health education short videos.

According to the Information Adoption Model, the influence of information cues on information adoption is mainly realized through perceived usefulness as a value judgment mechanism (Salehi-Esfahani et al., 2016). Information quality is an important antecedent of perceived usefulness, while perceived usefulness plays a key mediating role between information cues and information adoption (Cheung et al., 2008; Erkan and Evans, 2016). Previous research has also shown that, in social media use and interpersonal communication contexts, information quality can significantly enhance users’ perceived usefulness, which further has a positive effect on their adoption of social relationship information and travel information (Tang et al., 2026). Therefore, in the context of AI-generated health education short videos, high-quality information is more likely to be regarded by users as helpful and valuable, thereby enhancing their perceived usefulness.

In addition, information quality may also influence users’ trust judgments toward AI-generated content. Research on online health information has shown that users’ initial trust in health information is influenced by perceived information quality, and perceived information quality is one of the important factors in forming trust in health information (Mun et al., 2013). Based on this, in the context of AI-generated health communication, users may also judge whether AI-generated health information is reliable based on whether the content is accurate, relevant, and complete. In the context of generative AI services represented by ChatGPT, previous research has found that the relevance, credibility, usefulness, and intelligence of AI-generated content can significantly enhance users’ perceived trust (Ali et al., 2023). Therefore, in the context of AI-generated health education short videos, when the information content shows higher levels of accuracy, clarity, completeness, and professionalism, users are more likely to believe that the information can help them understand health issues and are also more likely to regard AI-generated content as reliable, credible, and dependable. Based on the above analysis, this study proposes the following hypotheses:

*H1*: Information quality positively influences users’ perceived usefulness of AI-generated health education short videos.

*H2*: Information quality positively influences users’ AI trust in AI-generated health education short videos.

#### 2.2.2 Source credibility

Source credibility refers to information recipients’ overall judgment of whether the information provider is trustworthy, professional, and reliable (Ayeh, 2015). In general, source credibility is mainly reflected in the expertise, trustworthiness, and authority of the source, and it is an important basis for individuals’ information adoption (Jin et al., 2016; Koo et al., 2014). In this study, source credibility specifically refers to users’ overall judgment of whether the publisher, presenting entity, and underlying information sources of AI-generated health education short videos are reliable, professional, and credible.

According to the Information Adoption Model, source credibility influences users’ evaluation of the value and usefulness of information (Le, 2023). When users perceive an information source as more reliable, they are more likely to regard the information as useful and worth referring to, thereby increasing their adoption intention (Erkan and Evans, 2016). Previous studies have shown that when users encounter information from different sources, they tend to trust and accept information from authoritative or highly credible sources (Hovland and Weiss, 1951; Kelman and Hovland, 1953; Lemire et al., 2008). In the context of health short videos, users’ perceptions of information credibility are jointly influenced by the creator’s professional background, content authority, and positive evaluations and interactive feedback on the platform. These factors further affect users’ judgments of usefulness, trust, and adoption intention (Han et al., 2025).

In the context of AI-generated health communication, source credibility not only influences users’ judgment of whether the information is useful but also affects their psychological evaluation of whether AI-generated content is trustworthy. Research on online health information has shown that source expertise is an important antecedent of users’ trust in health information. When users find it difficult to directly judge the professional accuracy of health information, they often rely on peripheral cues such as source expertise, authority, and credibility (Mun et al., 2013). Related research has also found that, in health information contexts, information published by physicians or professional entities and presented in an objective manner is more likely to receive higher credibility evaluations (Claggett et al., 2024). Therefore, when users perceive the publisher, presenting entity, or information basis of AI-generated health education short videos as more professional and reliable, they are more likely to regard the health information as helpful and to develop a higher level of AI trust. Based on the above analysis, this study proposes the following hypotheses:

*H3*: Source credibility positively influences users’ perceived usefulness of AI-generated health education short videos.

*H4*: Source credibility positively influences users’ AI trust in AI-generated health education short videos.

#### 2.2.3 Perceived usefulness

Perceived usefulness refers to the extent to which an individual believes that using a system, technology, or information can improve task performance, decision efficiency, or practical benefits (Davis, 1989). In general, perceived usefulness is regarded as an important variable linking information cues to adoption outcomes, reflecting individuals’ overall judgment of whether information is helpful and valuable as a reference (Sussman and Siegal, 2003). In this study, perceived usefulness specifically refers to the extent to which users believe that AI-generated health education short videos can help them understand health issues, make health-related judgments, and support health decision-making.

According to the Information Adoption Model, external cues such as information quality and source credibility influence users’ cognitive evaluation of information value and further affect information adoption intention (Erkan and Evans, 2016). Previous studies have shown that, in the context of health short videos, when users believe that the content helps them acquire health knowledge, understand health risks, or improve daily health management, they are more likely to develop a higher level of information adoption intention (Han et al., 2025). In the context of AI health information, users’ perceptions of the practical help and application value of generative AI are also important factors that promote their acceptance and use (Davis, 1989).

Furthermore, perceived usefulness not only influences whether users are willing to adopt information but may also participate in the formation of trust in AI-generated content (Ayeh, 2015). Existing studies on AI acceptance have suggested that perceived usefulness can promote users’ trust in AI because users’ positive evaluations of AI’s practical value and functional effectiveness strengthen their judgments of AI’s capability, reliability, and dependability (Khoso et al., 2025). It can therefore be inferred that, in the context of AI-generated health education short videos, when users believe that the information can provide valuable health explanations, reduce health uncertainty, and support health decision-making, they are more likely to regard AI-generated content as reliable, credible, and dependable. In other words, perceived usefulness may serve as an important cognitive mechanism linking information value evaluation, AI trust, and information adoption intention. Based on the above analysis, this study proposes the following hypotheses:

*H5*: Perceived usefulness positively influences users’ information adoption intention toward AI-generated health education short videos.

*H6*: Perceived usefulness positively influences users’ AI trust in AI-generated health education short videos.

#### 2.2.4 AI trust

AI trust refers to users’ overall judgment of the reliability, credibility, and dependability of AI systems or AI-generated information (Glikson and Woolley, 2020). Users’ trust in AI performance and its outputs is an important prerequisite for their acceptance and adoption. In contrast, doubts about the reliability of AI functions, mechanisms, or outputs may reduce users’ adoption intention (Esmaeilzadeh, 2020; Stevens and Stetson, 2023). In this study, AI trust refers to users’ overall judgment of whether AI-generated health education short videos are reliable, credible, and dependable in the processes of content generation, information provision, and health communication. It should be noted that AI trust in this study does not refer to users’ complete multidimensional evaluation of the overall capabilities of AI systems. Rather, it specifically refers to users’ trust judgments regarding the reliability, trustworthiness, and dependability of the health information presented in AI-generated health education short videos. Therefore, this study focuses on information trust in the context of AI-generated health information, rather than on comprehensive trust in AI systems that includes dimensions such as competence, transparency, predictability, and interpretability.

In the context of AI health information, AI trust is especially important for users’ adoption judgments (Reinhardt et al., 2026). Previous studies have shown that as AI systems become more autonomous, trust plays an increasingly important role in users’ acceptance and use of AI (Al Shboul et al., 2024; Alanezi, 2024). In health contexts, when AI provides information, health advice, or diagnostic support, users’ trust in AI often becomes an important factor influencing their acceptance (Huo et al., 2022). At the same time, research on generative AI health information has also shown that users’ trust in health information provided by AI is closely related to their willingness to further use and adopt such information (Svestkova et al., 2025). This suggests that when users perceive AI-generated health information as highly reliable and credible, they are more likely to form positive information adoption judgments. It can therefore be inferred that, in the context of AI-generated health education short videos, AI trust can be regarded as a key psychological mechanism in users’ information adoption decisions. The more users trust the reliability, professionalism, and dependability of AI-generated health information, the more likely they are to adopt such health education information. Based on the above analysis, this study proposes the following hypothesis:

*H7*: AI trust positively influences users’ information adoption intention toward AI-generated health education short videos.

#### 2.2.5 Health anxiety

Health anxiety refers to individuals’ excessive worry about illness, infection, or bodily sensations, as well as the amplified interpretation of bodily signals (Brown et al., 2020; Wang et al., 2024). Studies have shown that individuals with higher health anxiety usually pay more attention to health-related issues, are more likely to frequently seek online health information, and often regard information seeking as a reassuring behavior and coping strategy (Singh et al., 2016). In this study, health anxiety specifically refers to the persistent worry, vigilance, and unease that users bring to their exposure to AI-generated health education short videos regarding their own health problems, bodily sensations, or potential disease risks.

In digital health information environments, health anxiety can not only increase users’ attention to health-related content but also strengthen their need for information about symptom interpretation, disease risk, and health coping strategies (Muse et al., 2012). Research on health information adoption in short-video contexts has found that chronic health anxiety is positively associated with health information adoption intention. This suggests that users with higher health anxiety may be more likely to develop an intention to adopt health information because of their stronger sense of health uncertainty and need for risk explanation (Svestkova et al., 2025). Therefore, health anxiety can be viewed as an important psychological motivation that drives users to engage with and adopt health information (McMullan et al., 2019; Starcevic and Berle, 2013). However, the effect of health anxiety on health information behavior is not unidirectional. Excessive health anxiety may also lead to information avoidance, skepticism, or overinterpretation of health risks. Thus, health anxiety does not necessarily constitute a strong driver of information adoption, and its effect may depend on factors such as users’ age, health status, health literacy, and perceived source credibility.

Given that AI-generated health education short videos are easy to access, low in cost, and capable of providing rapid health explanations, this study argues that users with higher health anxiety may be more likely to pay attention to and refer to relevant health information. However, because health anxiety may also trigger skepticism or avoidance, this study treats it as an individual psychological factor that may influence information adoption intention, with an effect that may be weaker than information evaluation factors such as AI trust and perceived usefulness. Based on the above analysis, this study proposes the following hypothesis:

*H8*: Health anxiety positively influences users’ information adoption intention toward AI-generated health education short videos.

#### 2.2.6 The mediating roles of perceived usefulness and AI trust

According to the Information Adoption Model, external information cues usually do not directly translate into information adoption intention; instead, they need to pass through users’ cognitive evaluation of information value (Cheung et al., 2008; Erkan and Evans, 2016; Sussman and Siegal, 2003). In the classic IAM framework, information quality and source credibility represent content cues and source cues, respectively, while perceived usefulness is regarded as an important mediating variable linking information cues to information adoption (Cheung et al., 2008; Erkan and Evans, 2016; Jin et al., 2016). In the context of AI-generated health education short videos, information quality and source credibility may influence not only users’ judgment of the usefulness of health information but also their evaluation of the reliability, credibility, and dependability of AI-generated content. Previous research has shown that users’ trust in online health information is influenced by factors such as information quality, source expertise, and perceived risk (Mun et al., 2013). At the same time, AI trust is also an important psychological variable for explaining users’ acceptance and adoption of AI-generated information (Glikson and Woolley, 2020; Liu and Sun, 2025; Stevens and Stetson, 2023). Therefore, information quality and source credibility may further influence users’ information adoption intention through perceived usefulness and AI trust.

In addition, perceived usefulness and AI trust may have a sequential psychological relationship. Previous studies have indicated that perceived usefulness not only affects users’ information adoption and technology acceptance but may also strengthen users’ trust in AI systems or AI-generated content. This is because users’ positive evaluations of AI’s practical value and functional effectiveness help them form judgments about AI’s capability, reliability, and dependability (Ali et al., 2023; Davis, 1989; Khoso et al., 2025). In the present research context, when users believe that AI-generated health education short videos can help them understand health issues, reduce health uncertainty, or support health decision-making, they are more likely to develop trust in AI-generated content and ultimately increase their information adoption intention. In other words, users’ adoption of AI-generated health information may follow a chained psychological process of “usefulness evaluation–AI trust formation–adoption intention.” Based on the above analysis, this study proposes the following hypotheses:

*H9a*: Perceived usefulness mediates the relationship between information quality and information adoption intention.

*H9b*: AI trust mediates the relationship between information quality and information adoption intention.

*H9c*: Perceived usefulness and AI trust have a chain-mediating effect between information quality and information adoption intention.

*H10a*: Perceived usefulness mediates the relationship between source credibility and information adoption intention.

*H10b*: AI trust mediates the relationship between source credibility and information adoption intention.

*H10c*: Perceived usefulness and AI trust have a chain-mediating effect between source credibility and information adoption intention.

## 3 Research design

Based on the conceptual model described above, this study used partial least squares structural equation modeling (PLS-SEM) to examine the research model and hypotheses, with the aim of identifying factors associated with users’ information adoption intention toward AI-generated health education short videos and their relative predictive roles. PLS-SEM is suitable for testing models that include multiple latent variables and their relationships, and it provides a rigorous approach for hypothesis testing and model evaluation (Jain et al., 2023). PLS-SEM is mainly used to examine the path relationships in a theoretical model and their statistical significance. However, relying solely on path coefficients may not fully capture the relative importance of different predictors in a prediction task (Bellary et al., 2024). Prior studies have suggested that artificial neural networks (ANN) can serve as a complementary predictive method to SEM and can be used to assess the relative importance of input variables for outcome variables (Xu et al., 2023). In addition, importance–performance map analysis (IPMA) can further identify factors with practical optimization value by simultaneously examining the importance and performance level of each variable based on path analysis results (Garg et al., 2025).

Therefore, the combined use of PLS-SEM, ANN, and IPMA in this study is not a simple repetition of analyses, but is based on their complementary functions. PLS-SEM is mainly used to test the path relationships in the theoretical model and their significance. ANN is used as a complementary predictive analysis tool to examine whether the importance ranking of significant predictors identified in PLS-SEM remains stable within a data-driven predictive framework. It should be noted that the ANN models in this study are local prediction models constructed based on the PLS-SEM results. Each model contains only a small number of input variables and one output variable. Therefore, ANN analysis is not interpreted as complex deep learning modeling, nor is it used to claim the discovery of complex nonlinear mechanisms that PLS-SEM cannot identify. Its main role is to provide supplementary and confirmatory evidence for the importance ranking of key predictors. On this basis, IPMA is further used to identify priority variables for improvement from the two dimensions of “importance” and “performance.” Thus, the three methods serve theoretical testing, predictive robustness checking, and practical implications, respectively.

The analysis process consisted of four steps. First, SPSS was used to conduct descriptive statistical analysis to clarify the basic characteristics of the sample. Second, SmartPLS was used to assess the reliability and validity of the measurement model and to evaluate the relationships among latent variables in the structural model. Third, based on the PLS-SEM results, direct predictors that reached statistical significance were included in the ANN models, and the neural network module in SPSS was used to assess the relative importance of each variable within a nonlinear predictive framework. Finally, IPMA was conducted based on the PLS-SEM results to identify key variables that had high importance for information adoption intention toward AI-generated health education short videos but still had room for improvement in performance.

### 3.1 Sample selection and data collection

Before formal data collection, this study was reviewed and approved by the Ethics Committee of Sichuan University of Arts and Science, with the approval number SCWLLL20260027. All research procedures followed the principles of voluntary participation, informed consent, anonymity, and confidentiality. An informed consent statement was presented on the first page of the questionnaire, clearly explaining the study purpose, response requirements, data use, anonymity, and the right to withdraw. Participants were required to confirm their informed consent before proceeding to the formal questionnaire.

The questionnaire was designed using the Tencent Questionnaire platform and adopted a seven-point Likert scale. To ensure that respondents clearly understood the scoring criteria, the questionnaire stated at the beginning that the response scale ranged from 1 (“strongly disagree”) to 7 (“strongly agree”). To ensure that respondents had a relatively consistent understanding of the research object, this study provided a unified explanation of “AI-generated health education short videos” before the formal measurement items. In the questionnaire, AI-generated health education short videos were defined as health knowledge videos produced with the involvement of generative artificial intelligence technologies, including but not limited to AI digital human narration, AI-synthesized voice, AI-generated animation, AI-generated images, AI-assisted editing, or AI-assisted health education content. Such videos are typically used to introduce everyday health knowledge, disease prevention, lifestyle advice, or health management information. To help respondents understand the research object, the questionnaire provided a scenario description and reference materials before formal responses. Respondents were asked to answer based on their own understanding and viewing experience of AI-generated health education short videos. Therefore, this study focused on users’ overall perceptions, trust, and adoption intention toward health information in AI-generated health education short videos, rather than their evaluation of a single video, platform, or account.

A pilot test was conducted before the formal survey to examine the preliminary reliability and applicability of the questionnaire items. A total of 58 valid responses were obtained in the pilot test. The results showed a KMO value of 0.847, and Bartlett’s test of sphericity was significant, χ^2^(210) = 1712.622, *p* < 0.001, indicating that the data were suitable for factor analysis (Jiang et al., 2024; Liu et al., 2026). The communalities showed that the extraction values of all items were above 0.50, suggesting that the items could be well explained by the common factors (Peterson, 2000). The reliability analysis showed that the overall Cronbach’s α for the 18 core items was 0.961, and the Cronbach’s α values for each variable ranged from 0.929 to 0.972. These results indicate that the questionnaire had good internal consistency and was suitable for the formal survey (Jiang et al., 2025; Tang et al., 2026).

Based on these results, the study proceeded to formal data collection. During the formal survey, 519 questionnaires were collected. After removing incomplete responses and responses with identical answers throughout, 476 valid questionnaires remained, yielding an effective response rate of 91.7%. According to the “10-times rule” for PLS-SEM, the sample size exceeded 10 times the maximum number of measurement indicators and the maximum number of incoming paths in the model, meeting the minimum sample size requirement for PLS-SEM analysis (Leguina, 2015).

### 3.2 Variable measurement

To ensure the validity of the measurement scale, the questionnaire in this study was mainly developed based on mature scales from prior studies. First, measurement items that have been widely used in the existing literature were adopted to ensure that the dimensions and items for each variable had a solid theoretical basis. Then, in line with the research context of AI-generated health education short videos, some items were adapted to fit the specific setting. During this adaptation process, the core meaning of the original scales was retained as much as possible, while only the target object, technological context, and wording were adjusted to better match the research object and application scenario of this study. Among these items, the AI trust items mainly measured users’ judgments about the reliability, trustworthiness, and dependability of the health information presented in AI-generated health education short videos. AI-generated health education short videos were measured as an overall object, without further distinguishing among different forms of AI generation. After expert review and pilot testing, the formal questionnaire was finalized, including six variables and 18 measurement items. The variables and their corresponding measurement items are presented in Table 1.

**Table 1:** Questionnaire dimensions and specific content.

Dimension	Description	Sources
Information Quality	When watching AI-generated health education short videos, I can usually clearly understand the health information conveyed in them. AI-generated health education short videos usually provide relatively comprehensive health information. The health information in AI-generated health education short videos is usually clearly expressed and well organized.	Erkan and Evans (2016) and Tang et al. (2026)
Source Credibility	I usually consider the publishers or accounts of AI-generated health education short videos to be credible. I believe that the publishers or accounts of AI-generated health education short videos usually have a certain level of health-related knowledge. In my view, the publishers or accounts of AI-generated health education short videos usually have a certain degree of authority.	Komendantova et al. (2024) and Tang et al. (2026)
Perceived Usefulness	AI-generated health education short videos help me obtain useful health knowledge. AI-generated health education short videos help me better understand certain health issues. AI-generated health education short videos allow me to access health education information more conveniently.	Tang et al. (2026) and Yu et al. (2025)
AI Trust	I usually trust the health information provided by AI-generated health education short videos. I believe that the health information in AI-generated health education short videos is usually based on credible evidence. Overall, I have a relatively high level of trust in the health information presented in AI-generated health education short videos.	Svestkova et al. (2025)
Health Anxiety	I often worry that I may have some kind of health problem. I usually worry that I am at high risk of developing a serious disease. I frequently pay attention to pain, discomfort, or unusual sensations in my body.	Lagoe and Atkin (2015)
Information Adoption Intention	I am willing to accept the health information in AI-generated health education short videos. When needed, I will use the health information in AI-generated health education short videos as a reference. When the health information in AI-generated health education short videos is relevant to my actual situation, I am willing to consider it as a reference for health-related decision-making.	Komendantova et al. (2024), Tang et al. (2026), and Yu et al. (2025)

## 4 Results

### 4.1 Descriptive statistical analysis

The basic demographic information of the sample is presented in Table 2. A total of 476 valid questionnaires were obtained in this study. Among the respondents, 213 were male, accounting for 44.7%, and 263 were female, accounting for 55.3%. In terms of age distribution, respondents were mainly concentrated in the 18–24 age group, accounting for 59.9%. Regarding educational background, respondents with a bachelor’s or associate degree accounted for the largest proportion, at 71.0%. These results indicate that the sample in this study was generally skewed toward younger and more highly educated online users. Therefore, the findings are more applicable to users with relatively high exposure to digital media and should not be directly generalized to the broader public across all age groups, educational backgrounds, or health conditions.

**Table 2:** Basic demographic information.

Characteristics	Categories	*N*	%
Gender	Male	213	44.7
	Female	263	55.3
Age group	18 ~ 24	285	59.9
	25 ~ 30	75	15.8
	31 ~ 40	84	17.6
	41 ~ 50	18	3.8
	50 years and above	14	2.9
Education	High school or below/Vocational secondary school	42	8.8
	Bachelor’s or associate degree	338	71.0
	Master’s or doctoral degree	96	20.2

### 4.2 Common method bias test

Because all variables in this study were collected from self-reported questionnaire data at a single time point, it was necessary to control for and examine the risk of common method bias. First, in terms of procedural control, this study used an anonymous questionnaire to collect data. The first page of the questionnaire explained the research purpose, data use, anonymity, and voluntary participation principles, so as to reduce respondents’ evaluation concerns and social desirability bias. In addition, before the formal measurement items, this study provided a unified explanation of the concept of AI-generated health education short videos and the response criteria. During data cleaning, incomplete questionnaires and invalid questionnaires with identical responses across all items were removed.

Second, in terms of statistical testing, this study used the full collinearity VIF method to diagnose common method bias. The results showed that the VIF values of the predictors in the structural model ranged from 1.017 to 2.368, all below the recommended threshold of 3.3. This indicates that the model did not show serious full collinearity and also suggests that common method bias was unlikely to have a serious effect on the structural model results (Kock, 2015; Yuting and Rashid, 2025). It should be noted that, because this study used a cross-sectional self-reported questionnaire design, the risk of common method bias cannot be completely ruled out. Therefore, this issue is further acknowledged in the limitations section. Future research should use multi-source data, time-lagged designs, or behavioral data for further validation.

### 4.3 SEM-based analysis of the information adoption intention model for AI-generated health education short videos

#### 4.3.1 Measurement model assessment

To examine the fit between the theoretical model and the collected data, this study first assessed the reliability and validity of the measurement model. Because PLS-SEM was used for model testing, the reflective measurement model was evaluated mainly in terms of indicator reliability, internal consistency reliability, convergent validity, and discriminant validity.

For indicator reliability, this study assessed the outer loadings of each observed variable. As shown in Table 3, the factor loadings of all measurement items ranged from 0.837 to 0.955, exceeding the recommended threshold of 0.70. This indicates that each item explained its corresponding latent variable well and that the measurement indicators had good indicator reliability (Moussaïd et al., 2013; Sobti, 2019).

**Table 3:** Reliability and convergent validity of the measurement model.

Constructs	Items	Factor loadings	Cronbach’s α	CR	AVE
Information quality	IQ1	0.837	0.884	0.928	0.813
	IQ2	0.929			
	IQ3	0.936			
Source credibility	SC1	0.919	0.916	0.947	0.856
	SC2	0.926			
	SC3	0.931			
Perceived usefulness	PU1	0.938	0.938	0.960	0.889
	PU2	0.953			
	PU3	0.938			
AI trust	AIT1	0.955	0.949	0.967	0.908
	AIT2	0.950			
	AIT3	0.954			
Health anxiety	HA1	0.905	0.874	0.922	0.797
	HA2	0.883			
	HA3	0.890			
Information adoption intention	IAI1	0.925	0.928	0.954	0.874
	IAI2	0.944			
	IAI3	0.936			

For internal consistency reliability, Cronbach’s α and composite reliability (CR) were used. The results showed that Cronbach’s α values ranged from 0.874 to 0.949, and CR values ranged from 0.922 to 0.967. All values were above the recommended standard of 0.70, indicating high internal consistency reliability for each construct (Cheung et al., 2024; Izah et al., 2023; Liu et al., 2026). Although some constructs showed relatively high CR values, suggesting strong consistency among their items, the overall results remained within an acceptable range.

For convergent validity, average variance extracted (AVE) was used. As shown in Table 3, the AVE values of all latent variables ranged from 0.797 to 0.908, exceeding the recommended threshold of 0.50. This indicates that each construct explained a large proportion of the variance in its measurement items, supporting good convergent validity (Cheung et al., 2024).

For discriminant validity, this study first examined cross-loadings. The results in Table 4 show that each measurement item loaded higher on its corresponding construct than on other constructs, indicating that the items could adequately distinguish among different latent variables. In addition, the Fornell–Larcker results in Table 5 show that the square root of the AVE for each construct was greater than its correlations with other constructs.

**Table 4:** Cross-loadings of measurement items.

Item	IQ	SC	PU	AIT	HA	IAI
IQ1	**0.837**	0.456	0.549	0.431	0.071	0.453
IQ2	**0.929**	0.618	0.617	0.636	0.044	0.600
IQ3	**0.936**	0.601	0.637	0.617	0.036	0.609
SC1	0.620	**0.919**	0.662	0.730	0.118	0.671
SC2	0.558	**0.926**	0.641	0.647	0.136	0.636
SC3	0.555	**0.931**	0.611	0.706	0.119	0.664
PU1	0.615	0.682	**0.938**	0.738	0.133	0.755
PU2	0.642	0.650	**0.953**	0.709	0.121	0.711
PU3	0.635	0.619	**0.938**	0.696	0.115	0.720
AIT1	0.594	0.706	0.718	**0.955**	0.113	0.798
AIT2	0.591	0.699	0.724	**0.950**	0.082	0.803
AIT3	0.619	0.744	0.724	**0.954**	0.088	0.835
HA1	0.047	0.100	0.123	0.077	**0.905**	0.151
HA2	0.046	0.138	0.089	0.095	**0.883**	0.125
HA3	0.051	0.124	0.133	0.093	**0.890**	0.162
IAI1	0.593	0.664	0.742	0.810	0.163	**0.925**
IAI2	0.590	0.668	0.720	0.800	0.125	**0.944**
IAI3	0.557	0.661	0.705	0.780	0.176	**0.936**

Bold values indicate the loadings of each item on its corresponding construct.

**Table 5:** Discriminant validity.

Construct	IQ	SC	PU	AIT	HA	IAI
IQ	**0.901**					
SC	0.626	**0.925**				
PU	0.669	0.690	**0.943**			
AIT	0.631	0.752	0.758	**0.953**		
HA	0.054	0.134	0.131	0.099	**0.893**	
IAI	0.621	0.711	0.773	0.853	0.165	**0.935**

The bold diagonal values represent the square root of AVE, and the lower triangle shows the Pearson correlations.

To further assess discriminant validity, this study additionally reported the HTMT results. As shown in Table 6, most HTMT values among the constructs were below the recommended threshold of 0.90. The HTMT value between AI trust and information adoption intention was 0.908, slightly above 0.90. However, its 95% bootstrapping confidence interval was 0.868–0.941 and did not include 1, indicating that the two constructs still demonstrated acceptable discriminant validity.

**Table 6:** HTMT results.

Construct	IQ	SC	PU	AIT	HA	IAI
IQ						
SC	0.687					
PU	0.733	0.743				
AIT	0.679	0.804	0.803			
HA	0.063	0.151	0.142	0.109		
IAI	0.679	0.770	0.828	0.908	0.182	

Conceptually, AI trust reflects users’ evaluative judgment of the reliability, trustworthiness, and dependability of AI-generated health information, whereas information adoption intention reflects users’ behavioral tendency to accept, refer to, and use such health information. Although the two constructs are highly correlated, they correspond to two different stages: trust evaluation and behavioral intention. Therefore, based on the cross-loadings, the Fornell–Larcker criterion, and the HTMT results, the measurement model in this study demonstrated generally acceptable discriminant validity.

Overall, the measurement model met acceptable standards for indicator reliability, internal consistency reliability, convergent validity, and discriminant validity. This suggests that the measurement quality of the latent variables was satisfactory and that the structural model could be further tested.

#### 4.3.2 Structural model assessment

The structural model was assessed using the bootstrapping procedure in SmartPLS, with 5,000 resamples and a 95% confidence interval. The path coefficients (β), t statistics, *p* values, and hypothesis testing results are presented in Table 7. The structural model path testing results are shown in Figure 2. For the direct effects, information quality had a significant positive effect on perceived usefulness (*β* = 0.389, *t* = 8.763, *p* < 0.001) and AI trust (*β* = 0.105, *t* = 2.446, *p* = 0.014), supporting H1 and H2. Source credibility also had significant positive effects on perceived usefulness (*β* = 0.447, *t* = 10.824, *p* < 0.001) and AI trust (*β* = 0.404, *t* = 7.693, *p* < 0.001), supporting H3 and H4. Perceived usefulness significantly and positively affected information adoption intention (*β* = 0.289, *t* = 5.551, *p* < 0.001) and AI trust (*β* = 0.409, *t* = 7.819, *p* < 0.001), supporting H5 and H6. AI trust had the strongest significant effect on information adoption intention (*β* = 0.627, *t* = 12.106, *p* < 0.001), supporting H7. Health anxiety also had a significant positive effect on information adoption intention (*β* = 0.066, *t* = 2.881, *p* = 0.004), supporting H8, although its path coefficient was relatively small.

**Table 7:** Hypothesis testing results of the structural model.

Hypothesis	Path	*β*	Standard deviation	*T* statistics	*p* values	Supported
H1	IQ → PU	0.389	0.044	8.763	<0.001	Yes
H2	IQ → AIT	0.105	0.043	2.446	0.014	Yes
H3	SC → PU	0.447	0.041	10.824	<0.001	Yes
H4	SC → AIT	0.404	0.053	7.693	<0.001	Yes
H5	PU → IAI	0.289	0.052	5.551	<0.001	Yes
H6	PU → AIT	0.409	0.052	7.819	<0.001	Yes
H7	AIT→IAI	0.627	0.052	12.106	<0.001	Yes
H8	HA → IAI	0.066	0.023	2.881	0.004	Yes
H9a	IQ → PU → IAI	0.112	0.025	4.499	<0.001	Yes
H9b	IQ → AIT→IAI	0.066	0.027	2.443	0.015	Yes
H9c	IQ → PU → AIT→IAI	0.100	0.020	4.989	<0.001	Yes
H10a	SC → PU → IAI	0.129	0.025	5.207	<0.001	Yes
H10b	SC → AIT→IAI	0.253	0.038	6.696	<0.001	Yes
H10c	SC → PU → AIT→IAI	0.115	0.022	5.165	<0.001	Yes

β represents the standardized path coefficient. Values of *p* < 0.05 were considered statistically significant.

**Figure 2:**
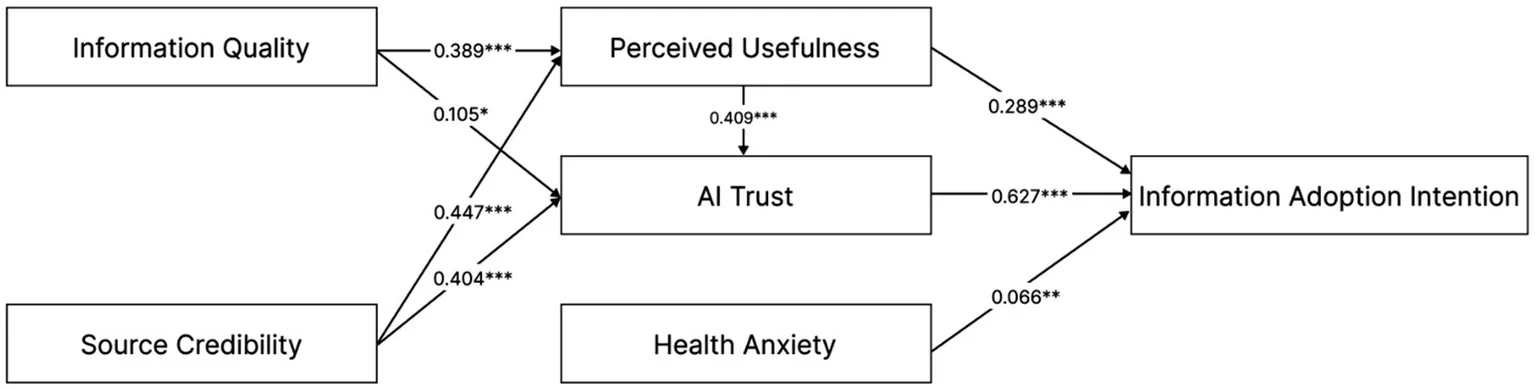
Structural model results.

For the mediation effects, perceived usefulness significantly mediated the relationship between information quality and information adoption intention (*β* = 0.112, *t* = 4.499, *p* < 0.001). AI trust also significantly mediated the relationship between information quality and information adoption intention (*β* = 0.066, *t* = 2.443, *p* = 0.015). In addition, the chain-mediating effect of perceived usefulness and AI trust between information quality and information adoption intention was significant (*β* = 0.100, *t* = 4.989, *p* < 0.001). Thus, H9a, H9b, and H9c were supported. Similarly, perceived usefulness significantly mediated the relationship between source credibility and information adoption intention (*β* = 0.129, *t* = 5.207, *p* < 0.001). AI trust also significantly mediated the relationship between source credibility and information adoption intention (*β* = 0.253, *t* = 6.696, *p* < 0.001), and the chain-mediating effect of perceived usefulness and AI trust between source credibility and information adoption intention was significant (*β* = 0.115, *t* = 5.165, *p* < 0.001). Therefore, H10a, H10b, and H10c were supported.

Overall, all hypothesized paths reached statistical significance, and H1 through H10c were supported. Among the direct effects, AI trust had the strongest effect on information adoption intention (*β* = 0.627), while health anxiety had the weakest effect on information adoption intention (*β* = 0.066), although it remained statistically significant. Among the mediation effects, the indirect effect of source credibility on information adoption intention through AI trust was the strongest (*β* = 0.253), and all other mediation paths were also significant.

To further assess the relative contribution of each predictor to the explanatory power of the endogenous variables, this study additionally calculated the *f*^2^ effect size. According to commonly used criteria, *f*^2^ values of 0.02, 0.15, and 0.35 represent small, medium, and large effects, respectively (Yao and Chiang, 2025). The results showed that AI trust had the largest effect size on information adoption intention (*f*^2^ = 0.725), reaching the level of a large effect. This further indicates that AI trust is a core explanatory variable for users’ information adoption intention. Perceived usefulness showed a medium effect on information adoption intention (*f*^2^ = 0.153), whereas health anxiety had a relatively low effect size (*f*^2^ = 0.018), below the commonly used threshold for a small effect. This suggests that although health anxiety was statistically significant, its actual explanatory contribution was relatively limited. In addition, information quality (*f*^2^ = 0.213) and source credibility (*f*^2^ = 0.282) both showed medium effects on perceived usefulness. Source credibility (*f*^2^ = 0.242) and perceived usefulness (*f*^2^ = 0.225) also showed medium effects on AI trust, whereas the effect size of information quality on AI trust was relatively low (*f*^2^ = 0.017).

To assess the explanatory and predictive power of the model, this study further calculated the coefficient of determination (*R*^2^) and the predictive relevance indicator (*Q*^2^) to evaluate the overall explanatory effect of the exogenous variables on the endogenous variables. As shown in Table 8, the *R*^2^ values for perceived usefulness, AI trust, and information adoption intention were 0.568, 0.680, and 0.769, respectively. These results indicate that the corresponding exogenous variables explained 56.8, 68.0, and 76.9% of the variance in the three endogenous variables, respectively. Overall, the model showed good explanatory power for all three endogenous variables, with the strongest explanatory power for information adoption intention.

**Table 8:** *R*^2^ and *Q*^2^ values of endogenous constructs.

Construct	*R* ^2^	*R*^2^ (adjusted)	*Q*^2^ predict
PU	0.568	0.567	0.565
AIT	0.680	0.678	0.604
IAI	0.769	0.768	0.558

In addition, the adjusted *R*^2^ values of the endogenous variables were close to their corresponding *R*^2^ values, indicating that the explanatory power of the model was relatively stable. In terms of predictive relevance, the *Q*^2^ predict values for perceived usefulness, AI trust, and information adoption intention were 0.565, 0.604, and 0.558, respectively, all of which were greater than 0. This suggests that the model had good predictive relevance (Kugara, 2026). Taken together, the structural model developed in this study demonstrated both strong explanatory power and good predictive ability.

### 4.4 SEM–ANN-based analysis of information adoption intention toward AI-generated health education short videos

To further examine the relative contribution of significant predictors in the complementary predictive analysis and to determine whether the importance rankings of key variables were consistent with the PLS-SEM results, this study introduced artificial neural networks (ANN) based on the PLS-SEM results and developed a hybrid SEM–ANN analytical model, as shown in Figure 3. According to the endogenous variables in the structural model, three ANN submodels were established. Model A used information adoption intention (IAI) as the output variable, with perceived usefulness (PU), AI trust (AIT), and health anxiety (HA) as input variables. Model B used AI trust (AIT) as the output variable, with information quality (IQ), source credibility (SC), and perceived usefulness (PU) as input variables. Model C used perceived usefulness (PU) as the output variable, with information quality (IQ) and source credibility (SC) as input variables. All input variables were derived from significant direct predictive paths identified in the PLS-SEM results.

**Figure 3:**
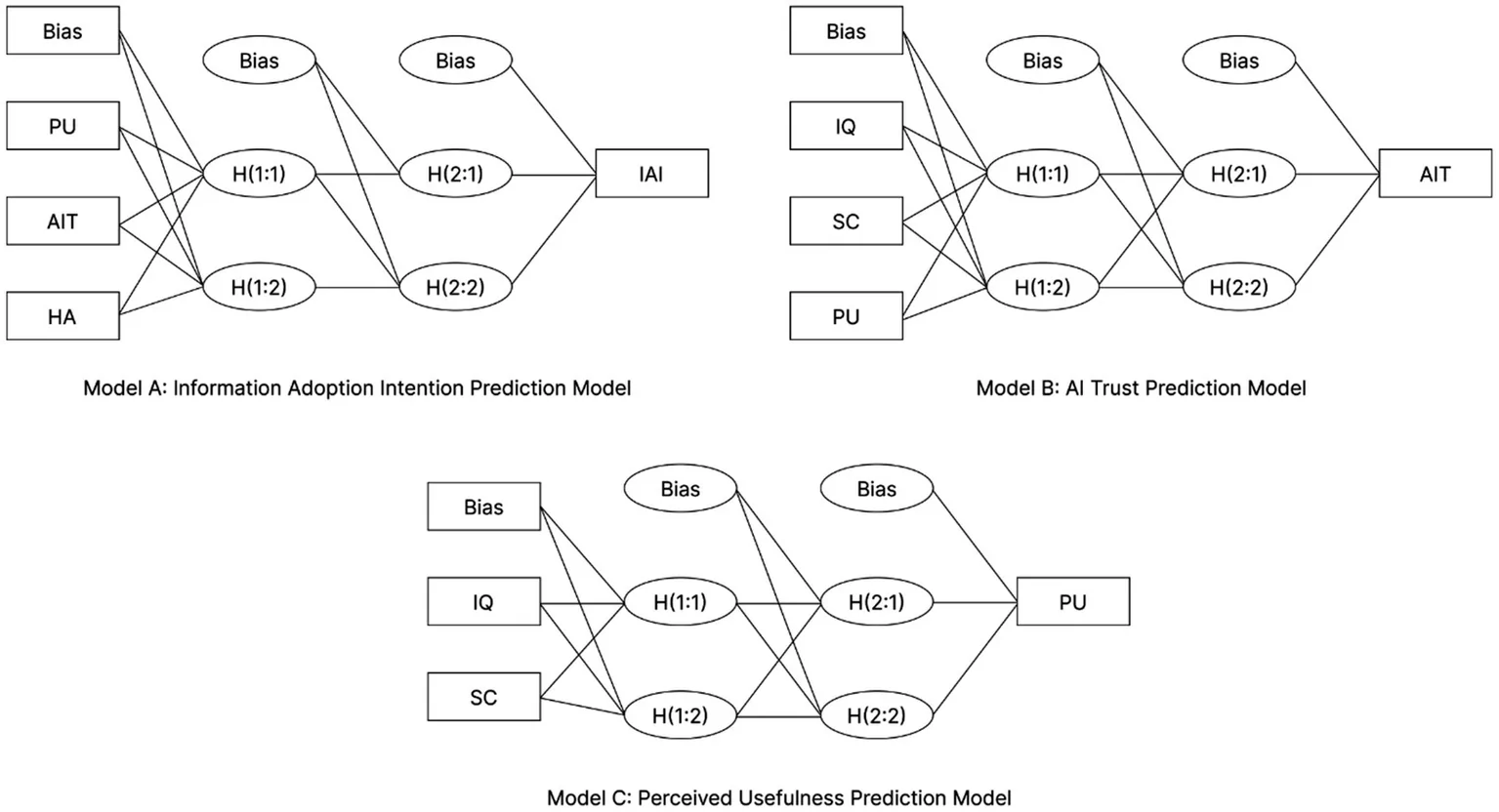
Feedforward neural network model.

This study used a multilayer perceptron (MLP) neural network model, and all variables were normalized to the [0, 1] range. To improve the stability of the results and reduce the risk of overfitting, each model was trained and tested using 10 random training–testing splits. In each split, approximately 90% of the sample was used for training and approximately 10% was used for testing. Model predictive performance was assessed using the root mean square error (RMSE), as shown in Table 9. The average training RMSE values for Models A, B, and C were 0.087, 0.097, and 0.102, respectively, while the average testing RMSE values were 0.087, 0.095, and 0.092, respectively. The relatively close RMSE values between the training and testing sets across the three models indicate that the ANN models showed good predictive stability and no clear evidence of overfitting.

**Table 9:** Predictive performance of the ANN models based on RMSE.

Neural network	Model A		Model B		Model C	
	Training	Testing	Training	Testing	Training	Testing
ANN1	0.085	0.084	0.094	0.119	0.101	0.091
ANN2	0.097	0.102	0.099	0.092	0.110	0.083
ANN3	0.085	0.072	0.096	0.091	0.102	0.084
ANN4	0.090	0.093	0.096	0.098	0.103	0.093
ANN5	0.084	0.095	0.100	0.090	0.104	0.072
ANN6	0.089	0.067	0.097	0.085	0.100	0.110
ANN7	0.084	0.079	0.096	0.111	0.102	0.093
ANN8	0.086	0.101	0.097	0.081	0.097	0.123
ANN9	0.085	0.086	0.097	0.102	0.102	0.091
ANN10	0.085	0.093	0.096	0.083	0.102	0.080
Mean	0.087	0.087	0.097	0.095	0.102	0.092
SD	0.004	0.012	0.002	0.012	0.003	0.015

The sensitivity analysis results are presented in Table 10. In Model A, AI trust (AIT) had the highest normalized importance (100.00%), followed by perceived usefulness (PU, 57.16%), whereas health anxiety (HA) had the lowest importance (10.21%). This indicates that AI trust was the most important input variable in the ANN prediction model. In Model B, perceived usefulness (PU) showed the highest normalized importance (100.00%), followed by source credibility (SC, 88.93%), while information quality (IQ) had a lower importance value (30.78%). These results suggest that perceived usefulness and source credibility were the main predictors of AI trust. In Model C, the normalized importance values of information quality (IQ) and source credibility (SC) were 100.00 and 92.01%, respectively, indicating that both were important predictors of perceived usefulness, with information quality showing slightly higher importance than source credibility.

**Table 10:** Results of sensitivity analysis.

ANN	Model A: Predicting IAI			Model B: Predicting AIT			Model C: Predicting PU	
	PU	AIT	HA	IQ	SC	PU	IQ	SC
ANN1	0.354	0.602	0.044	0.150	0.353	0.497	0.523	0.477
ANN2	0.443	0.535	0.023	0.157	0.395	0.449	0.565	0.435
ANN3	0.347	0.579	0.073	0.119	0.406	0.475	0.510	0.490
ANN4	0.266	0.644	0.090	0.180	0.365	0.455	0.564	0.436
ANN5	0.325	0.622	0.052	0.153	0.428	0.419	0.532	0.468
ANN6	0.268	0.640	0.093	0.056	0.441	0.503	0.477	0.523
ANN7	0.336	0.596	0.067	0.159	0.390	0.451	0.477	0.523
ANN8	0.400	0.557	0.043	0.151	0.421	0.428	0.501	0.499
ANN9	0.328	0.611	0.061	0.133	0.461	0.405	0.520	0.480
ANN10	0.348	0.588	0.064	0.143	0.387	0.469	0.539	0.461
Average relative importance	0.342	0.597	0.061	0.140	0.405	0.455	0.521	0.479
Normalized relative importance (%)	57.16	100.00	10.21	30.78	88.93	100.00	100.00	92.01

To further compare the PLS-SEM path coefficients with the ANN variable importance results, this study compared the importance rankings of the input variables in each model. The results are shown in Table 11. In Model A, the rankings produced by the two methods were fully consistent. AI trust was identified as the most important factor in predicting information adoption intention (PLS-SEM *β* = 0.627; ANN = 100.00%), followed by perceived usefulness (*β* = 0.289; ANN = 57.16%), while health anxiety showed the lowest importance (*β* = 0.066; ANN = 10.21%). In Model B, the rankings from PLS-SEM and ANN were also consistent. Perceived usefulness was the primary predictor of AI trust (*β* = 0.409; ANN = 100.00%), followed by source credibility (*β* = 0.404; ANN = 88.93%), with information quality ranking third (*β* = 0.105; ANN = 30.78%).

**Table 11:** Comparison of PLS-SEM and ANN results.

Model	Path	Path coefficient (PLS-SEM)	Normalized relative importance (%) (ANN)	Ranking based on PLS-SEM	Ranking based on ANN
Model A: Predicting IAI	AIT → IAI	0.627	100.00%	1	1
	PU → IAI	0.289	57.16%	2	2
	HA → IAI	0.066	10.21%	3	3
Model B: Predicting AIT	PU → AIT	0.409	100.00%	1	1
	SC → AIT	0.404	88.93%	2	2
	IQ → AIT	0.105	30.78%	3	3
Model C: Predicting PU	SC → PU	0.447	92.01%	1	2
	IQ → PU	0.389	100.00%	2	1

In Model C, the rankings differed slightly between the two methods. The PLS-SEM results showed that the path coefficient from source credibility to perceived usefulness was slightly higher than that from information quality to perceived usefulness (*β* = 0.447 vs. *β* = 0.389). In contrast, the ANN results showed that information quality had the highest normalized relative importance (100.00%), followed by source credibility (92.01%). Overall, PLS-SEM and ANN produced consistent rankings in Models A and B. Although a slight difference was observed in Model C, both methods indicated that information quality and source credibility were important predictors of perceived usefulness. These results suggest that the ANN analysis in this study did not produce findings that were substantially different from the PLS-SEM results. Instead, it validated the stability of the importance rankings of key variables from the perspective of complementary predictive analysis. Therefore, the ANN results mainly provide predictive robustness evidence for the PLS-SEM results rather than proposing a new nonlinear theoretical mechanism.

### 4.5 Importance–performance map analysis (IPMA)

To further identify practical priorities for improving users’ information adoption intention, this study conducted an importance–performance map analysis (IPMA) using SmartPLS. IPMA simultaneously considers the total effect of each antecedent variable on the target construct and its performance level. Importance reflects the overall influence of a variable on the target construct, whereas performance indicates the actual level of that variable in the sample (Garg et al., 2025). In general, variables with high importance but relatively low performance should receive priority for improvement.

In this study, information adoption intention (IAI) was used as the target construct, and its direct antecedents—perceived usefulness, AI trust, and health anxiety—were selected for IPMA. The results are shown in Table 12. AI trust had the highest importance (total effect = 0.627), but its performance value was only 46.356. Therefore, it ranked first in priority, indicating that AI trust is the most important factor to address when improving users’ information adoption intention. Perceived usefulness ranked second in importance (total effect = 0.545), with a performance value of 57.394, suggesting that it also has a strong influence on information adoption intention but currently performs relatively well. Health anxiety showed the lowest importance (total effect = 0.066), with a performance value of 47.947, and ranked third in priority.

**Table 12:** IPMA results for information adoption intention.

Construct	Importance/Total effect	Performance	Priority
AIT	0.627	46.356	1
PU	0.545	57.394	2
HA	0.066	47.947	3

Overall, AI trust had the highest importance, but its performance level was lower than that of perceived usefulness. This indicates that AI trust is a priority area for improvement, as it shows high importance while still leaving room for further enhancement. In comparison, perceived usefulness also had relatively high importance, but its current performance was comparatively better. Health anxiety had the lowest importance and therefore should not be regarded as the primary target for practical intervention. Accordingly, improving users’ trust in AI-generated health information should serve as a key entry point for optimizing information adoption intention. In practice, this can be achieved by strengthening professional review, clearly identifying information sources, explaining the degree of AI involvement, and adding risk reminders, thereby improving users’ ability to evaluate the content and enhancing its perceived credibility.

## 5 Discussion

### 5.1 The central role of AI trust in information adoption

This study found that AI trust was the most critical factor influencing users’ adoption of information from AI-generated health education short videos. The PLS-SEM results showed that AI trust had the strongest positive effect on information adoption intention. The ANN sensitivity analysis further indicated that AI trust had the highest normalized relative importance in predicting information adoption intention. The IPMA results also showed that AI trust had the highest importance but a relatively low performance level. Taken together, these findings suggest that, in the context of AI-generated health education short videos, users’ willingness to adopt relevant health information largely depends on whether they perceive AI-generated content as reliable, credible, and dependable.

This finding is consistent with the conclusions of Guo et al. and Svestkova et al., who found that users’ trust in generative AI health information and in the reliability of AI performance is an important factor influencing their willingness to adopt or use such information (Guo et al., 2025; Svestkova et al., 2025). This further suggests that information adoption in AI-generated health education short videos differs from the acceptance of information in conventional health short videos. Health information itself has a relatively high-risk nature and may influence users’ judgments about disease, physical conditions, and health behaviors. When health information is also generated with the involvement of AI, users need not only to judge whether the information is helpful, but also to further assess whether AI-generated content is reliable (Dunn et al., 2023). Therefore, users’ information adoption process is not merely a matter of content acceptance, but also involves a comprehensive trust judgment concerning AI technology, information sources, and health-related risks.

This result also extends the explanatory power of the Information Adoption Model in the context of AI health communication. The traditional IAM emphasizes the roles of information quality, source credibility, and perceived usefulness in information adoption. However, this study shows that, in the context of AI-generated health content, trust judgment has a more prominent psychological role. Even when users perceive the content as practically useful, their adoption intention may still be constrained if they lack trust in AI-generated content. Therefore, AI trust can be regarded as an important psychological mechanism linking information evaluation and adoption intention.

### 5.2 The chain mechanism of perceived usefulness and AI trust

The results showed that perceived usefulness not only had a significant positive effect on information adoption intention, but also significantly and positively influenced AI trust. It also mediated the relationships between information quality, source credibility, and information adoption intention. This finding is consistent with the basic logic of the Information Adoption Model, which suggests that information cues such as information quality and source credibility affect users’ information adoption through perceived usefulness. It also supports the view of Sussman and Siegal that perceived usefulness mediates the relationship between influence processes and information adoption (Sussman and Siegal, 2003). This indicates that the Information Adoption Model is also applicable to the context of AI-generated health education short videos. When users believe that AI-generated health education short videos can help them acquire health knowledge, understand health issues, or support health-related judgments, they are more likely to develop a stronger intention to adopt the information.

Furthermore, this study found that perceived usefulness also contributes to the formation of AI trust. This finding is consistent with Khoso et al.’s research on AI acceptance. Khoso et al. noted that perceived usefulness can promote users’ trust in AI because positive evaluations of AI’s practical value and functional effectiveness strengthen users’ judgments of AI capability, reliability, and dependability (Khoso et al., 2025). In the present context, users do not trust AI in an abstract sense. Rather, they are more likely to perceive AI-generated health information as reliable and dependable after recognizing its practical value. This suggests that, in the context of AI-generated health education short videos, “usefulness” and “trustworthiness” are closely connected. Perceived usefulness is not only a direct antecedent of information adoption, but also an important cognitive basis for the formation of AI trust.

The chain mediation results further showed that information quality and source credibility can influence information adoption intention through the pathway of “perceived usefulness → AI trust.” This finding extends the traditional IAM pathway of “information cues–perceived usefulness–information adoption” into a chained psychological process of “information cues–perceived usefulness–AI trust–information adoption.” In other words, users’ information adoption may involve a sequential psychological process: they first judge whether the information is useful based on content and source cues, then develop trust in AI-generated content based on this usefulness evaluation, and finally decide whether to adopt the relevant health information. Thus, this study further enhances the applicability of the IAM in the context of AI health communication.

### 5.3 The differential roles of information quality, source credibility, and health anxiety

This study found that both information quality and source credibility significantly and positively influenced perceived usefulness, indicating that content cues and source cues are important bases for users’ evaluation of the value of AI-generated health education short videos. This result is consistent with the findings of Pu Han et al., who found that perceived usefulness, information quality, and perceived trust significantly and positively influenced users’ intention to adopt health short videos (Han et al., 2025). This study further shows that, in the context of AI-generated health education short videos, users are more likely to perceive content as valuable when the video presents information clearly, completely, and in a well-organized manner. Similarly, when the publisher or account is perceived as professional, authoritative, and credible, users are more likely to regard the related content as useful.

Notably, when predicting AI trust, source credibility played a much stronger role than information quality, while the direct effect of information quality on AI trust was relatively weak. This finding is consistent with the conclusions of Eastin and Pian et al. Eastin showed that source expertise affects users’ credibility judgments of online health information, especially when users encounter unfamiliar health information, in which case source cues become an important basis for judgment (Eastin, 2001). Pian et al. also found that users consider whether online health information is credible, safe, and professional when evaluating its quality (Pian et al., 2022). Therefore, in the context of AI-generated health information, users’ trust in AI does not come solely from the content itself, but also depends strongly on their judgment of the information source. For ordinary users, external cues such as the publisher’s identity, account qualifications, professional endorsement, and authoritative sources may be important bases for forming AI trust.

In addition, health anxiety was significantly and positively associated with information adoption intention, but the strength of this association was relatively weak. This result is generally consistent with research on health information adoption and health anxiety. Xuan et al. found that chronic health anxiety was positively associated with health information adoption intention, suggesting that health anxiety may increase individuals’ need for health explanations and risk-coping information, thereby making them more willing to adopt relevant health information (Xuan et al., 2026). However, the effect of health anxiety on health information behavior is not unidirectional. It may also lead to information avoidance, skepticism, or overinterpretation of health risks. The results of this study showed that health anxiety had a small path coefficient and a low *f*^2^ effect size, which was below the commonly used threshold for a small effect. This indicates that its actual explanatory contribution was limited. Therefore, health anxiety is better understood as an auxiliary individual psychological factor and should not be interpreted as a main driver of users’ adoption of information from AI-generated health education short videos. This result may also be related to the sample characteristics of this study, which mainly included young and highly educated online users. This group usually has relatively high exposure to digital media and relatively low health risk, which may limit the role of health anxiety in this sample.

### 5.4 Theoretical and practical implications

This study has several theoretical implications. First, it focuses on AI-generated health education short videos as a new form of health communication that combines video communication and AI-generated content. In doing so, it expands the research scope of digital health communication and health information adoption. Previous studies have mainly focused on conventional health short videos, online health information, or text-based generative AI health tools. By incorporating AI-generated health education short videos into the information adoption research framework, this study helps deepen the understanding of health information adoption behavior in the era of generative AI.

Second, this study introduces AI trust and health anxiety into the IAM and reveals the trust mechanism and individual psychological factors involved in the adoption of AI-generated health information. The results show that AI trust not only directly influences information adoption intention, but also mediates the relationship between perceived usefulness and information adoption intention. This indicates that, in the context of AI health communication, the traditional IAM pathway of “information cues–usefulness–adoption” needs to be extended into a psychological process that includes AI trust. At the same time, the significant role of health anxiety suggests that users’ adoption of AI-generated health information is influenced not only by information cues and AI trust judgments, but also by their own health concerns and risk sensitivity.

Finally, this study combines PLS-SEM, ANN, and IPMA as complementary methods for theoretical path testing, supplementary predictive validation, and practical priority identification. PLS-SEM was used to test the theoretical paths among variables. ANN was used to verify the stability of the importance ranking of key variables from a complementary predictive perspective. IPMA further identified AI trust as the primary target for improving information adoption intention. This methodological combination helps provide a more comprehensive understanding of the psychological processes related to the adoption of information from AI-generated health education short videos.

At the practical level, the findings suggest that improving users’ adoption intention toward AI-generated health education short videos requires not only enhancing the usefulness of the content but also strengthening users’ trust in AI-generated health information. According to the IPMA results, AI trust had the highest importance but still showed room for improvement in performance, and should therefore be treated as a priority for practical optimization. Content producers should ensure that health information is accurate, clear, complete, and easy to understand, and should avoid exaggerated titles, vague suggestions, or unverified health claims. For high-risk content related to disease prevention, symptom judgment, and medication advice, the scientific basis, scope of applicability, and medical consultation reminders should be clearly stated.

At the same time, platforms and publishers should strengthen source credibility. Platforms can help users evaluate the reliability of information sources by labeling publisher qualifications, displaying review information from physicians or medical institutions, providing references, and indicating review dates and target audiences. For health education short videos generated with AI involvement, the degree of AI participation should be explained, such as whether the content involves AI-assisted editing, AI-synthesized voice, AI digital human narration, or content mainly generated by AI. It should also be noted that transparent disclosure does not mean that more information is always better. Future research still needs to examine how different levels, placements, and wording of disclosure affect user trust.

For users with higher health anxiety, AI-generated health education short videos may provide some explanatory and reassuring functions, but inappropriate content may also amplify their anxiety. Therefore, platforms and content producers should not use health anxiety to create fear-based communication. Instead, they should help users form reasonable information judgments through clear explanations, rational suggestions, risk reminders, and medical consultation guidance.

## 6 Conclusion

This study focused on AI-generated health education short videos. Based on the Information Adoption Model, it incorporated AI trust and health anxiety to construct and test the psychological mechanisms related to users’ information adoption intention. The results showed that both information quality and source credibility were significantly and positively associated with users’ perceived usefulness, and were further related to AI trust and information adoption intention. Perceived usefulness and AI trust played important mediating roles between information quality, source credibility, and information adoption intention. This suggests that users’ adoption of AI-generated health education short videos is not simply the result of information exposure, but involves a psychological process of “information cue evaluation–perceived usefulness–AI trust formation–adoption intention.”

The study further found that AI trust was the most critical factor associated with users’ information adoption intention. AI trust showed the highest importance in both the PLS-SEM path analysis and the ANN complementary predictive analysis. The IPMA results also showed that AI trust had the highest importance but a relatively low performance level, indicating that improving users’ trust in AI-generated health information should be a priority for strengthening their information adoption intention.

In addition, health anxiety had a significant but relatively weak positive effect on information adoption intention. This suggests that users with higher health anxiety may be more likely to pay attention to and refer to information in AI-generated health education short videos, but their adoption behavior is still mainly influenced by AI trust and perceived usefulness. Therefore, AI health communication should not simply make use of users’ health concerns. Instead, it should help users form rational and reliable information judgments through high-quality content, credible sources, transparent disclosure, and professional review mechanisms.

This study has several limitations. First, it used cross-sectional questionnaire data. Although such data can be used to examine associations among variables, they do not allow strong causal inference. For example, this study found significant associations among information quality, source credibility, perceived usefulness, and AI trust. However, these findings do not mean that higher information quality necessarily leads to higher AI trust. It is also possible that users who already trust the relevant information source or AI-generated content may evaluate its information quality more positively. Future research could use experimental designs or longitudinal methods to further examine the causal mechanisms among information quality, source credibility, AI trust, and information adoption.

Second, this study measured users’ information adoption intention through self-reported questionnaires rather than actual adoption behavior. Therefore, there remains a gap between subjective intention and real behavior. Future research could incorporate platform-based behavioral data or experimental behavioral indicators, such as clicks, viewing time, saves, shares, account following, clicks on authoritative source links, medical consultations, or actual changes in health behavior, to further examine the effects of AI-generated health education short videos on real health information adoption and health behavior.

Third, this study examined AI-generated health education short videos as an overall category and did not further distinguish among different forms of AI generation. For example, AI digital human narration, AI-synthesized voice, AI-generated animation, AI-assisted editing, and fully AI-generated content may have different effects on users’ trust, cognitive load, and information adoption. Future research could further compare how different AI presentation forms and degrees of AI generation influence users’ psychological responses.

Fourth, AI trust in this study mainly measured users’ judgments about the reliability and trustworthiness of AI-generated health information. It did not fully cover the multidimensional structure of AI trust, such as competence, transparency, predictability, interpretability, and perceived human oversight. Future research could adopt a more comprehensive multidimensional AI trust scale and further include factors such as perceived risk, algorithm literacy, health literacy, privacy concerns, and human–AI interaction experience to develop a more complete model of AI-based health communication adoption.

Fifth, the sample in this study was skewed in terms of age and education level, which may limit the external validity of the findings. Respondents aged 18–24 accounted for a relatively high proportion of the sample, and respondents with an associate or bachelor’s degree also accounted for a large share. Therefore, the findings more strongly reflect the perceptions and adoption intentions of young, highly educated online users. This group usually has greater exposure to digital media and higher familiarity with technology, and may also have relatively lower health risks. Their AI trust, health anxiety, and information adoption judgments may differ from those of older adults, patients with chronic diseases, or individuals with lower health literacy. Future research could broaden the sample and compare groups with different ages, health conditions, education levels, and health literacy to examine the external validity and cross-group applicability of the model.

Overall, based on a sample mainly composed of young and highly educated online users, this study extends the application of the Information Adoption Model to the context of AI-generated health communication and highlights the central role of AI trust in users’ adoption of information from AI-generated health education short videos. The findings provide practical implications for the production of AI-based health education content, platform governance, and public health communication. They also offer empirical evidence for understanding the psychological mechanisms of health information adoption in the era of generative AI.

## Data Availability

The raw data supporting the conclusions of this article will be made available by the authors, without undue reservation.
